# Impact of a school-based health intervention program on body composition among South African primary schoolchildren: results from the KaziAfya cluster-randomized controlled trial

**DOI:** 10.1186/s12916-021-02223-x

**Published:** 2022-01-27

**Authors:** Kurt Z. Long, Johanna Beckmann, Christin Lang, Harald Seelig, Siphesihle Nqweniso, Nicole Probst-Hensch, Ivan Müller, Uwe Pühse, Peter Steinmann, Rosa du Randt, Cheryl Walter, Jürg Utzinger, Markus Gerber

**Affiliations:** 1 Swiss Tropical and Public Health Institute, University of Basel, Socinstrasse 57, 4051 Basel, Switzerland; 2 Department of Sport, Exercise and Health, University of Basel, Basel, Switzerland; 3 Department of Human Movement Science, Nelson Mandela University, Port Elizabeth, South Africa

**Keywords:** Physical activity, Micronutrients, Randomized trial, School-age children, South Africa

## Abstract

**Background:**

The prevalence of overweight and obesity is increasing among African children potentially predisposing them to greater obesity and non-communicable diseases (NCDs) in adulthood. This risk may be higher among growth-impaired children who may have greater fat mass. Therefore, we examined the effects of school-based physical activity (PA) promotion and multi-micronutrient supplementation (MMNS) on body composition among South African children enrolled in a longitudinal school-based randomized controlled trial.

**Methods:**

Children were cluster-randomized by class to one of four groups: (a) a physical activity group (PA), (b) a multi-micronutrient supplementation group (MMNS), (c) a physical activity + multi-micronutrient supplementation group (PA + MMNS), and (d) control group, and were being followed for 3 years. Linear random effects regression models with random intercepts for school classes tested the associations of each intervention arm with overall fat mass (FM), fat-free mass (FFM), truncal fat mass (TrFM), and truncal fat-free mass (TrFFM) at 9 months (T2) for boys and girls. These differences were then explored among children who differed in height velocity (HV).

**Results:**

A total of 1304 children (614 girls, 667 boys) in twelve clusters were assessed at baseline and after 9 months follow-up (T2). At baseline, approximately 15% of children were classified as overweight or obese while approximately 38% of children were classified as mildly stunted or moderately/severely stunted. Among girls, promotion of PA was associated with reduced FM and TrFM at T2 while MMNS was associated with increased FFM. Children with reduced HV in the PA arm had reduced FM while children in the MMNS arm with lower HV had increased FM compared to children in the control arm. Similarly, children with lower HV in the MM and PA groups had reduced TrFM compared to children in the control arm.

**Conclusions:**

Our study suggests that the promotion of school-based physical activity programs and micronutrient supplementation can reduce childhood adiposity and so reduce the risk of obesity and chronic diseases later in adulthood.

**Trial registration:**

ISRCTN, ISRCTN29534081. Registered on August 9, 2018. The trial was designed, analyzed, and interpreted based on the CONSORT protocol (Additional file 1: CONSORT checklist for randomized trial)

**Supplementary Information:**

The online version contains supplementary material available at 10.1186/s12916-021-02223-x.

## Background

The prevalence of overweight, obesity, and non-communicable diseases (NCDs) has increased significantly in a number of regions of Sub-Saharan Africa in the last 30 years [1, 2]. Similar increases in overweight and obesity are occurring among African children which potentially predisposes them to greater obesity and NCDs in adulthood [3]. These trends are magnified by the simultaneous co-occurrence of both growth impairment and overweight or obesity in children and adults. Studies in several low- and middle-income countries (LMICs) have reported associations between impaired growth, obesity, and increased abdominal fat among adolescences and adults [4]. This form of the double burden of malnutrition (DBM) is prevalent in South Africa where childhood stunting and child overweight/obesity co-exist [5, 6].

The major drivers of obesity are reduced physical activity (PA) and the overconsumption of energy-dense, nutrient-poor foods and beverages in communities in LMICs passing through the nutrition transition where traditional diets high in cereal and fiber are replaced by more Western pattern diets high in sugars, fat, and animal-source food [7, 8]. Children recovering from malnutrition who are stunted have greater replenishment of body fat stores than lean body mass during catch-up growth [9, 10]. The overconsumption of high-fat, nutrient-poor foods that occurs with the nutrition transition, therefore, may result in even greater body fat mass gains among these children [11].

Vitamin and mineral deficiencies are associated with obesity and greater fat mass in adults and children and so may be an unrecognized biological mechanism contributing to trends in obesity and NCDs [12]. Preliminary supplementation trials have reported that carotenoids and carotenoid derivatives as well as zinc can reduce body mass index (BMI) *z*-scores and fat mass accrual among obese children and among stunted children [13, 14]. Addressing how deficiencies may contribute to body composition, childhood obesity, and long-term risk of obesity, and NCDs in adulthood in South African countries that are passing through the nutrition transition should be a priority in public health research.

Efforts to identify childhood obesity prevention interventions that are the most effective have increasingly focused on school-based PA promotion programs [15, 16]. Few school-based health interventions, however, have been carried out in African countries. The Disease, Activity and Schoolchildren’s Health (DASH) project carried out in disadvantaged primary schools in Gqeberha (formerly Port Elizabeth), South Africa, reported that increased PA levels are associated with lower risks of obesity and hypertension and resulted in significant decreases in children’s body mass index (Müller I, al. e: Effect of a multidimensional fitness education and hygieneintervention programme on physical fitness and body-mass-index in disadvantaged primary schoolchildren (DASH) in Port Elizabeth, South Africa: a longitudinal study, under review). These findings suggest that the promotion of PA intervention does have a positive effect on BMI and body composition in a South Africa context.

Further improvements in child health and body composition may be achieved through programs that simultaneously promote PA and micronutrient supplementation. The finding that supplementation can reduce BMI *z*-scores and fat mass accrual among obese children suggests that micronutrient status affects the development of obesity and obesity-related conditions through metabolic pathways that differ from those promoted through PA [13, 14]. Supplementation may also complement PA programs in communities where DBM is prevalent by improving body composition among growth-impaired children. These efforts may be further enhanced through the use of validated estimates of body composition among children when determining the outcomes of the study.

The present study reports on associations of PA promotion and MMNS with altered patterns of body composition among South African children enrolled in a longitudinal school-based cluster-randomized controlled trial. The specific objective of the study is to determine the effects of different intervention arms on body composition estimates during the critical period of pre-adolescent growth and development among these children. It specifically tests whether the individual and combined PA and MM interventions are associated with changes in overall and truncal estimates of fat mass (FM) and fat-free mass (FFM) and whether these effects differ among children with different growth velocities. The cluster-randomized design was used to reduce treatment contamination between the intervention arms and the control group. Greater FM and abdominal obesity in childhood partly a consequence of high caloric intake are associated with both overweight and obesity leading to increased risks of type 2 diabetes (T2D) and cardiovascular diseases in adulthood [17]. Increased FFM leads to greater basal metabolic rate and calorie expenditure and so can reduce the risk of fat accumulation as well as reduce insulin sensitivity [18]. It is important to investigate these body composition measures in parallel to determine how interventions may alter their relative balance and so reduce obesity risk. The results can aid in the development of more effective community-specific interventions that have the potential to reduce the burden of childhood obesity and long-term metabolic disease in Sub-Saharan countries that are rapidly urbanizing.

## Methods

### Participating schools and subjects

Children included in this analysis were part of a larger cluster-randomized trial assessing the effect of PA and MMNS on children’s growth, health, and well-being (the KaziAfya project) in three African countries [19]. Children were recruited in the South African component of the study from public schools in peri-urban neighborhoods of Gqeberha in the Eastern Cape Province that had poor ratings in terms of national poverty tables, income levels, dependency ratios, and literacy rates. Schools were then determined to be eligible if they had facilities to implement physical education lessons, if they were not involved in any other research projects, and were not in an area where government nutrition interventions were taking place during the study period.

Children aged between 6 and 12 years at baseline were eligible for inclusion in the project if they were attending grades 1 to 4; were not participating in any food/nutritional programs; were not suffering from clinical conditions, which prevent participation in PA; and not participating in other clinical trials. Children were excluded from data analyses (but not from the intervention) if they have a congenital or acquired alteration of the gastro-intestinal tract impairing absorption of the MMNS and had received regular vitamin and mineral supplements through food/nutritional programs in the past 6 months. Power calculations indicated that a total sample of 1096 children was needed per study site (calculations based on G*power 3.1: *f* = 0.10, alpha error probability = 0.05, power = 0.80, number of groups = 12, number of measurements = 3). The targeted sample size was increased to 1320 children per country at baseline assuming a yearly dropout rate of 10% with approximately 330 students assigned to one of the four intervention arms.

Oral consent was sought for each child’s participation in addition to written informed consent from the parents/guardians. Participation of children was voluntary, and so children could withdraw at any time without further obligations. Each child was assigned a unique identification number to ensure confidentiality. Children were then enrolled after the parent/ guardian granted written informed consent.

### Randomization to treatment groups

Participants were cluster-randomized by class to one of four groups using a random number table: (a) a physical activity group (PA), (b) a multi-micronutrient supplementation group (MMNS), (c) a physical activity + multi-micronutrient supplementation group (PA + MMNS), and (d) control group. Random allocation was carried out so that all four intervention arms were present in each school across the four grade levels to ensure balanced age groups. The project coordinator generated the random allocation sequence while the local project coordinator who was blinded to the multi-micronutrient groups assigned participants by classes to interventions and enrolled them.

Children allocated to the MMNS arm received a daily chewing tablet containing vitamins and trace elements based on the MixMe™ powder sprinkle developed by DSM Nutritional Products (Additional file 2: multi-vitamin mineral supplement tablets) [20] modified by replacing vitamin A with 4500 mg of β-carotene. β-Carotene was included in the supplement due to the previous findings of its efficacy in reducing obesity and abdominal fat [13]. Children allocated to the PA arm participated in daily in-class activity breaks as well as one weekly 45–60-min playful physical education lessons and one 45–60-min lessons involving dancing-to-music and improvised movements to music called Moving to Music, which were incorporated into the main school curriculum. All physical education and moving-to-moving lessons are described in detail as part of the KaziKidz toolkit (see: https://www.kazibantu.org/kazikidz/). Children allocated to the PA + MMNS group participated in the PA program and received the daily supplement.

Only teachers of the PA intervention classes received the teaching materials involving the Intervention Toolkit “KaziKidz” lesson plan for physical activity and Moving to Music to assure that there was no spillover effect with other classes. They were assisted by a physical education coach on a weekly basis to assure its proper implementation. Children allocated to MMNS or “placebo” conditions received the usual school lessons taught by their teachers. Children in the PA group and non-intervention arm received a placebo product with the same packaging and similar taste to the micronutrient supplement. This packaging ensured that children, teachers, and study personnel were blinded regarding the MMNS or placebo tablets. Teachers at school administered the MMNS and placebo tablets 5 days a week to the students in the different intervention arms and kept records of the administration of tablets.

### Body composition assessment and anthropometric measurements of children

Data assessments took place at children’s schools before children in the different intervention arms began receiving the treatment measures. The main assessments were performed across two school days with assessment procedures the same across all schools.

Body composition was assessed via bioelectrical impedance analysis (BIA) using a wireless body composition monitor (Tanita MC-580; Tanita Corp., Tokyo, Japan). Children wearing only light sports clothing were asked to stand barefoot on the metal plates of the machine, being guided by the research assistant to ensure optimal contact according to the device manufacturer’s instructions. The MC-580 was also used to assess body weight, to the nearest 0.1 kg. Body height was measured to the nearest 0.1 cm with each child standing with his/her back erect and shoulders against a stadiometer. Sex-specific height and weight-for-age and BMI *z*-scores were then computed from the CDC/WHO growth reference data [21]. Children with *z*-scores between − 1 SD > *z* > − 2 SD for height-for-age *z*-score (HAZ) were classified as mildly stunted, children with *z*-scores between − 2 SD > *z* > − 3 SD were classified as moderately stunted, and children with *z*-scores < − 3 SD were classified as severely stunted. Children were also classified using the CDC standards as underweight (below the 5th percentile), normal weight (5th percentile up to the 84th percentile), overweight (85th to 94th percentile), and obese (equal to or greater than the 95th percentile).

Information was collected from parents regarding the infrastructure and housing characteristics (house type, number of bedrooms, type of toilet and access to indoor water, indoor toilet/bathroom, and electricity) and related to ownership of three durable assets (presence of a working refrigerator, washing machine, and car). Socio-economic status (SES) categories were then constructed by dividing the sum of these household characteristics and durable assets by terciles across the entire sample.

Children were then assessed at 9-month T2 follow-up within each school with assessments performed simultaneously across two school days. Body composition and weight were again assessed using the BIA wireless body composition monitor, and height was measured using the stadiometer. All collected data was double entered in EpiData, validated, and merged into a single SPSS data file. Survey data assessed via paper and pencil questionnaire was double entered on Epidata.

### Statistical methods

The endpoints in this study were estimates of overall fat mass, lean body mass, truncal fat mass, and truncal lean body mass at the second follow-up. Descriptive statistics (M and SD) were first calculated to describe the characteristics of the sample by intervention group. ANOVA was first used to compare all groups simultaneously. Linear random effects regression models with random intercepts for school classes were then used to test the associations of the intervention arms coded as separate dummy variables with each of the separate overall and truncal body composition estimates at T2. Models were run for all children combined and separately for boys and girls, adjusting for each of the body composition estimates at baseline to control for the initial unequal means of the variables of interest across the intervention arms. A second set of analyses was then carried out that adjusted for children’s sex, HAZ at baseline, age at T2, and composite measure of household SES. HAZ was included in the model to adjust for the differences in fat mass and lean body mass that may occur as a result of the differing growth patterns.

Additional analyses were then carried out to further address how such differences in growth patterns may modify the associations of the interventions with the body composition estimates. As such, interaction terms between the dummy variable for each intervention group and height velocity (HV) were included in the adjusted model. When an interaction was found, results were then presented by strata of poor and good growth defined by a cutoff point for HV of < − 0.75 SD (poor growth < − 2.8 cm; adequate growth > − 2.8 cm) [22]. Interactions between the intervention groups and the child’s sex were also tested. Statistical significance was set at a probability level of *P* < 0.05 or *P* < 0.1 for interactions. A model for each of these outcomes was run with a school-specific random effect to measure the difference between the average score at each school and the average score for the whole sample. All statistical tests were performed using SPSS® 26 (IBM Corporation, Armonk, USA).

## Results

### Baseline characteristics of children

**Fig. 1:**
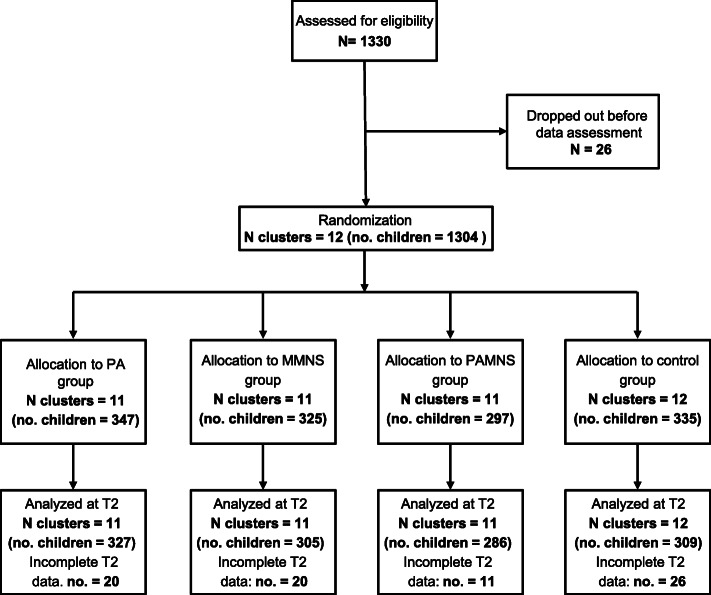
CONSORT flow diagram of children in the South African component of the KaziAfya project included in the analysis

A total of 1304 children were enrolled (following written parental informed consent) from quintile 3 public schools in Gqeberha in the South African component of the KaziAfya project and randomized to one of four groups by the twelve class clusters (Table 1, Fig. 1). Seventy-seven children were excluded due to missing values for outcome variables or independent/confounding variables at either T1 baseline (February to April 2019) or the T2 follow-up (July to September 2019) (Fig. 1). A total of 1227 children (586 girls, 641 boys) remained in the study: 327 in the PA group (167 girls, 160 boys; clusters = 11), 286 in the PA + MMNS group (140 girls, 146 boys; clusters = 11), 305 in the MM group (139 girls, 166 boys; clusters = 11), and 309 in the control group (140 girls, 169 boys; clusters = 12). A third and final post-intervention round (T3) was carried out from August to November 2021; the results of which will be reported in subsequent manuscripts.

**Table 1:** Child and household characteristics at baseline in Gqeberha, South African component of the KaziAfya project

By treatment group	Intervention group			
	Physical activity, *N* class clusters = 11 (*n* children = 347)	Physical activity + MMNS, *N* class clusters = 11 (*n* children = 297)	MMNS, *N* class clusters = 11 (*n* children = 325)	Placebo, *N* class clusters = 12 (*n* children = 335)
**Child characteristics**	*M (95% CI)*	*M (95% CI)*	*M (95% CI)*	*M (95% CI)*
Age at entry [means in years]	8.75 (8.65–8.94)	8.34 (8.18–8.50)	8.18 (8.02–8.33)	7.96 (7.81–8.11)
Boys [*n* (%)]	169 (49.4)	151 (51.2)	171 (53.6)	176 (54.2)
**Anthropometric measures at baseline**				
Height	127.38 (126.4–128.3)	124.96 (123.9–126.0)	123.71 (122.7–124.7)	122.67 (121.6–123.6)
Weight	27.33 (26.61–28.05)	25.55 (24.77–26.33)	24.82 (24.07–25.57)	23.76 (23.76–24.50)
BMI	16.45 (16.17–16.74)	16.13 (15.83–16.43)	16.05 (15.76–16.33)	15.83 (15.34–15.91)
**Categories of malnutrition at baseline**	*n (%)*	*n (%)*	*n (%)*	*n (%)*
Overweight or obese^a,b^	53 (16.6)	46 (16.3)	49 (16.0)	36 (12.4)
Stunted^c^	29 (9.1)	27 (9.5)	25 (8.1)	30 (9.3)
**Body composition**	*M (95% CI)*	*M (95% CI)*	*M (95% CI)*	*M (95% CI)*
Overall fat mass (kg)	6.66 (6.32–6.99)	5.89 (5.52–6.25)	5.85 (5.05–6.21)	5.33 (4.98–5.68)
Overall fat-free mass (kg)	20.67 (20.23–21.12)	19.66 (19.18–20.15)	18.96 (18.50–19.43)	18.44 (17.97–18.90)
Truncal fat mass (kg)	3.12 (12.96–3.28)	2.77 (2.60–2.94)	2.78 (2.63–2.95)	2.55 (2.40–2.71)
Truncal fat-free mass (kg)	13.00 (12.78–13.21)	12.56 (12.32–12.80)	12.27 (12.04–12.50)	12.01 (11.80–12.23)
**Overall SES index**^**d**^	*n (%)*	*n (%)*	*n (%)*	*n (%)*
Category 1	101 (29.2)	106 (35.7)	107 (32.9)	120 (35.8)
Category 2	150 (43.2)	131 (44.1)	134 (41.2)	137 (41.0)
Category 3	96 (27.7)	60 (20.2)	84 (25.8)	78 (23.3)

^a^Overweight: BMI *z*-score > + 1 SD (85th to 94th percentile)

^b^Obese: > + 2 SD (equal to or greater than the 95th percentile)

^c^Stunting: HAZ < − 2 SD

^d^*SES* socio-economic status. SES categories are constructed by dividing the sum of household characteristics and durable assets by terciles across the entire sample

There was a significant difference in children’s age across the intervention groups with children in the PA-MM group having higher mean age compared to children in the other groups (Table 1; *P*-value < 0.001). Significant differences were also found for children in height (*P*-value < 0.001), weight (*P*-value < 0.001), and BMI (*P*-value < 0.001), with the lowest means for all three measures found in the control group and the highest means found in the PA group. A similar pattern was observed for children’s overall FM (*P*-value < 0.001) and overall FFM (*P*-value < 0.001) with the lowest means found among children in the control group and the highest means found among children in the PA group. No differences occurred among children across groups for TrFM and TrFFM. Between 12 and 17% of children were classified as overweight or obese, and approximately 9% were classified as stunted with no difference across the groups. No differences were found in the distribution of children’s households across the SES categories with 30–35% of households classified in the lowest category, 40–44% in the intermediate category, and 20–27% in the highest.

### Effect of interventions on body composition outcomes at T2 follow-up

Promotion of PA was associated with increased overall FM, FFM, and TrFM among the 1227 children in the 12 clusters at T2 in unadjusted models. In models adjusted for age, SES, and body composition estimates and HAZ at baseline, promotion of PA was associated with reduced overall FM (*P*-value = 0.03) and TrFM (*P*-value < 0.01) among children at T2 (Table 2). In contrast, MMNS was associated with increased FFM (*P*-value < 0.01). These associations were found predominantly among girls when the analyses were carried out separately by sex (Table 3). As such, among girls, promotion of PA was associated with reduced FM (*P*-value = 0.02) and TrFM (*P*-value = 0.02) at T2 while MMNS was associated with increased FFM (*P*-value = 0.03). Boys in the PA + MMNS arm did have reduced TrFFM (*P*-value = 0.01). No associations were found among boys in the other intervention arms for the remaining body composition outcomes.

**Table 2:**
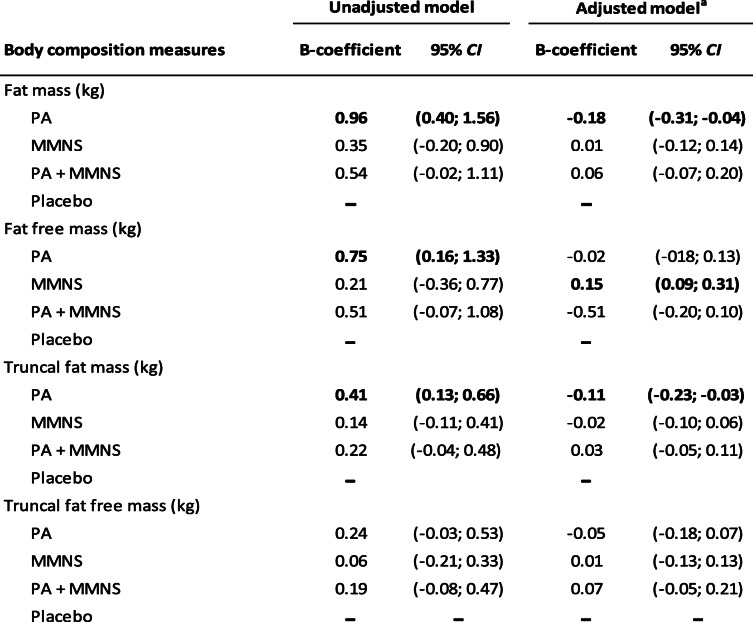
Body composition measures at first follow-up (T2) among children in Gqeberha, South African component of KaziAfya project by treatment group

*PA* physical activity group, *MMNS* multi-micronutrient supplement group, *PA + MMNS* physical activity group + multi-micronutrient group

^a^Models adjusted for age, sex, socio-economic status, and baseline body composition and HAZ

**Table 3:**
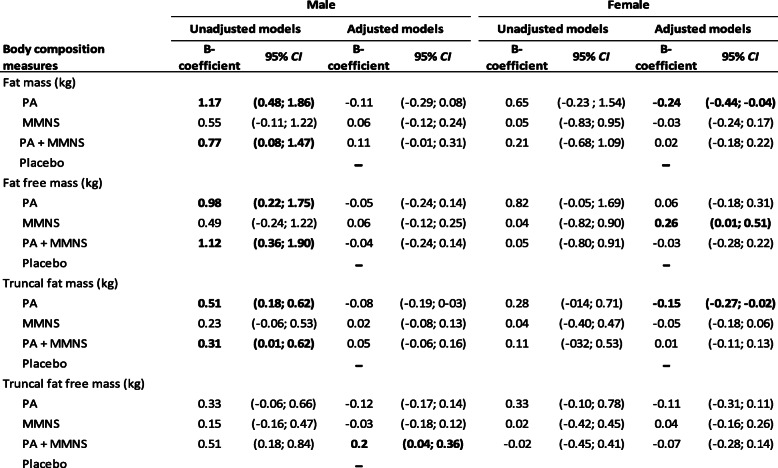
Body composition measures at first follow-up (T2) among boys and girls in Gqeberha, South African component of the KaziAfya project by treatment group and sex

The control group is used as a reference

*PA* physical activity group, *MMNS* multi-micronutrient supplement group, *PA + MMNS* physical activity group + multi-micronutrient group

^a^Models adjusted for age, socio-economic status, body composition measures, and HAZ at baseline

Analyses were then carried out to address how associations of the intervention arms with body composition outcomes differed by HV in the adjusted models. Significant interactions were found between PA and HV for FM (*B* coefficient = 0.12, CI 0.003–0.237, *P* for interaction = 0.04). Interactions were also significant between MMNS and HV for FFM (*B* coefficient = 0.30, CI 0.25–0.42, *P* for interaction = 0.01). The results were then stratified using a cutoff point for change in height of − 0.75 SD (≤ 2.8 cm; > 2.8 cm) to further determine how the differences in growth modified these associations. Children in the PA arm with HV below the cutoff point had reduced FM at T2 compared to children in the control group (Table 4, *P*-value < 0.001). In contrast, children with HV below this cutoff point in the MMNS arm had increased estimates of FFM compared to children in the control group (*P*-value = 0.3, Table 4). No associations were observed for any body composition estimates among children with HV above the cutoff point.

**Table 4:**
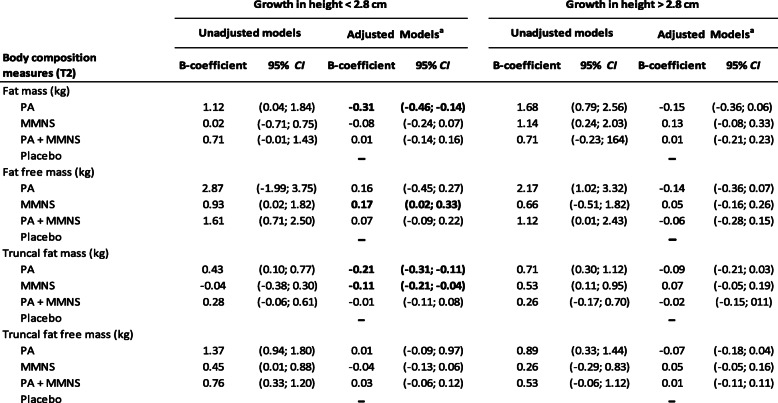
Overall and truncal body composition measures at first follow-up (T2) among children in Gqeberha, South African component of the KaziAfya project by treatment group and height velocity differences

The control group is used as a reference

*PA* physical activity group, *MMNS* multi-micronutrient supplement group, *PA + MMNS* physical activity group + multi-micronutrient group

^a^Models adjusted for age, sex, socio-economic status, and baseline measures of body composition and HAZ

Significant interactions were also identified between changes in height for TrFM among children in the PA and MMNS intervention arms (*B* coefficient = − 0.10, *P* for interaction = 0.01, and *B* coefficient = − 0.06, *P* for interaction = 0.01, respectively). No significant interactions were found between PA-MM and HV for TrFM. The analysis of associations of the intervention arms with TrFM was again stratified using the same cutoff point of HV of − 0.75 SD (≤ 2.8 cm; > 2.8 cm). Children in the MMNS and PA arms who had lower growth had reduced TrFM compared to children in the control group (Table 4, *P*-value = 0.001 and *P*-value = 0.04, respectively). No significant associations were found between intervention arms and estimates of TrFFM for children in the poorer growth strata. Additionally, no associations were found among children for any of the body composition estimates with height above this cutoff point.

### Adverse outcomes associated with the interventions

Adverse effects associated with the multi-micronutrient supplement were a concern since components of the MMNS such as iron have been reported to cause adverse reactions. However, in our study, only 18 students reported such adverse effects as diarrhea, vomiting, and nausea which were limited in duration and severity. No additional side effects have been reported in the follow-up.

### Trial registration

The intervention study was registered with the ISRCTN (Trial registry number: ISRCTN29534081, http://www.isrctn.com/ISRCTN29534081).

## Discussion

We have found that PA promotion and MMNS were associated with reduced fat mass and increased lean body mass, respectively, among South African children enrolled in a longitudinal school-based intervention trial based in peri-urban neighborhoods of Gqeberha. These effects were found predominantly among girls while minimal effects were found among boys. Children in the MMNS and PA arms who grew less in the follow-up period also had reduced FM and TrFM compared to children in the non-intervention arm. These distinct and contrasting effects of PA and MMNS related to growth patterns have not been reported before as far as we know. These findings need to be explored further given that some of the results are secondary outcomes. However, public health efforts to reduce DBM and non-communicable diseases in LMICs passing through the nutrition transition should continue to consider the promotion of PA in schools especially for girls, but should also consider micronutrient supplementation of children with reduced growth trajectories.

These findings are partially supported by previous intervention studies among school-age children. Observational studies have found that adults and children who reported being more physically active had lower BMI and a lower body fat percentage compared to those who were less physically active [23, 24]. However, a systematic review and meta-analysis examining the effects of school-based PA interventions on children’s BMI found that PA interventions did not improve BMI, although they had other beneficial health effects [15]. More recently, a school-based multi-component PA intervention carried out in Switzerland reported reduced adiposity among children who also had improved PA and fitness [25]. The DASH study also found favorable effects of PA intervention on body composition among children although this study used BMI and skinfold measures to estimate fat mass.

There are a number of studies examining the effects of MMNS on body composition among children. Studies examining the effects of supplementation when given prenatally or in early childhood have found variable or inconsistent results [26]. For example, the effect of Zn supplementation on body composition when given alone or in combination with other micronutrients may not be consistent [27]. However, it may have a beneficial effect on FFM among growth-impaired children [14]. Canas et al. [13] found that supplementation of obese children with mixed carotenoids reduced BMI *z*-scores and decreased the accrual of abdominal subcutaneous adipose tissue. The variability in these studies may be partly due to the use of anthropometric measures that indirectly determine body composition which complicates the efforts to accurately determine alterations in the FM and/or FFM components of the body.

Our results are able to more systematically address these relationships in a LMIC context using a multi-arm longitudinal design that compares and contrasts the effects of the different intervention arms. Additionally, the inclusion of β-carotene in the supplement may have been more effective in producing changes in the body composition than retinol or other forms of vitamin A. As a result, we have found that PA and MMNS have separate effects on body composition and that these effects are found predominantly among girls. Our findings that PA promotion was negatively associated with FM and TrFM in girls are only partially supported by smaller, cross-sectional studies. Thompson et al. [28], for example, reported that PA was negatively associated with percent FM only among Samoan boys while no similar effect was found among girls. However, carbohydrate intake was negatively associated with percent FM in girls only possibly due to the type of carbohydrate consumed in the traditional Samoan diet. Few studies have reported on sex differences of the associations between micronutrient intake and body composition measures that would be comparable to our results. These distinct effects and findings that children with reduced growth benefitted more from MMNS suggest PA and MMNS affect the body composition in different ways through distinct biological pathways.

It is important to identify what physiological mechanisms underlie these pathways in order to further understand and improve the effectiveness of these school-based interventions in promoting child health and reducing the risk of obesity and NCD. The regulation of adipose tissue by micronutrients may be one physiological mechanism underlying the effect of MMNS on body composition. Fat-soluble vitamins can modify fat mass through the regulation of adipocyte differentiation and metabolism [29]. Retinoic acid, β-carotene, and 25(OH)2D, the bioactive form of vitamin D, have shown anti-adipogenic effects through their activations of key transcription factors involved in adipocyte differentiation [30–32]. Vitamin C, vitamin E, and β-carotene deficiencies may alter the genetic expression of leptin important in regulating food intake energy expenditure and constancy of adipose tissue thus leading to leptin resistance and increased adiposity and obesity risk [33, 34]. Zinc repletion also leads to increased tissue leptin secretion, which may subsequently lead to reduced adipose tissue mass and so modify the risk of obesity [35].

The physiological mechanisms underlying the effect of PA on body composition may be through its effect on energy balance. Among adults, increased PA induces a negative energy balance and consequently reduced fat mass along as there is no increase in energy intake [36, 37]. Exercise-induced reduction in weight and fat mass corresponds with reduced adipocyte size and increased fat oxidation rather than the regulation of adipogenesis as seen with micronutrients [38]. PA promotion is associated with reductions in leptin and increases in adiponectin plasma concentrations, but these changes are not associated with changes in fat mass [39, 40].

A combined or additive effect of PA and MMNS on body composition was not found among children in our study. The contrasting effects of PA and MMNS described above may underlie the lack of an effect seen among children in this group. These effects could involve separate pathways and so may not act synergistically. Further research is needed to determine whether negative associations found between PA and FM estimates and obesity among children in industrialized countries and LMICs are due to reduced adipocyte size or regulation of adipogenesis [16, 41].

These underlying mechanisms may explain our findings within the context of poor child growth and adipose tissue development in LMICs. Deficiencies in micronutrients such as zinc can lead to poor growth [42] and may lead to increased adipogenesis. Additionally, growth impairment is associated with impairment of fat oxidation, which is a risk factor for excess weight gain and greater fat storage in adipose tissues [43–45]. Catch-up growth among malnourished children may lead to increased fat mass accrual as a result of impairment of physiological mechanisms regulating energy balance and fat oxidation [46, 47]. This may be accelerated in LMICs like South Africa passing through the nutrition transition where urban lower-income households are consuming energy-dense and nutrient-poor diets and where childhood stunting still persists [48]. The reduction in FM and TrFM among children in the MMNS arm with lower HV in our study may result from the anti-adipogenic effect of micronutrient repletion among deficient children. The universal effect of PA, in turn, may reflect its effect on energy balance, which may not be conditional on patterns of growth.

The age of children in our study is the period when the adiposity rebound occurs, the time at which BMI starts to rise after infancy. There is a clear relationship between the age at adiposity rebound and final adiposity with early adiposity rebound (age < 5.5 years) leading to higher adiposity level and obesity in later childhood and adolescence and a five-time greater risk of adult obesity [49, 50]. Studies have also reported that children who are overweight at 7 years old had a 400% increased risk of T2D as adults [51]. However, the latter study also reported that men who had been overweight at 7 years of age but had had remission of overweight by 13 years of age had a reduced risk of having T2D in adulthood similar to the risk among men who had never been overweight. These findings suggest that interventions that target communities with a high prevalence of poor child growth in LMICs could lead to a more favorable timing of the adiposity rebound and so lower adiposity levels and subsequent risk of adult obesity and NCDs.

Our findings have important clinical relevance on this timing and subsequent risk of adult obesity. The effect size of the reduction of FM found in our study among children in the PA and MMNS arms could be sufficient to lead to remission of overweight in adolescence and risk of adult obesity risk and T2D. These results if further confirmed can lead to the development of more effective interventions in a critical period of child health and development. Our results also suggest that intervention studies and public health programs concerned with preventing childhood obesity should consider a sex-specific approach.

A number of limitations of our study need to be considered. The results have limited generalizability since all children are from peri-urban settings and quintile 3 schools located in marginalized areas. A different picture might emerge in rural settings or in wealthier student populations across South Africa. Another limitation may be the omission of dietary data, which is certainly having an effect on body composition. Dietary intake was collected at baseline from all enrolled children using food frequency questionnaires. The validity of using the BIA estimates to assess the overall fat mass may be an additional limitation of the study since some researchers have criticized the use of these estimates [52]. Numerous studies have established that these estimates of body composition components are very accurate relative to methods such as dual-energy X-ray absorptiometry (DXA) [53] and have more biological relevance and context for readers. Longitudinal multivariate statistical models are now being developed that analyze the structural relationships between intervention arms and baseline dietary patterns. These models will provide a more accurate picture of the effects of the intervention arms on the associations between growth and body composition over time.

## Conclusions

Our findings have shown that PA is associated with reduced overall fat mass and TrFM while MMNS is associated with reduced FM and TrFM among children with reduced growth. These results suggest that such intervention strategies can be incorporated into double-duty approaches involving programs and policies that simultaneously tackle both nutrient deficiencies and overweight, obesity, and NCDs [54]. Such an approach may prove effective in reducing DBM in South Africa and other LMICs that are passing through the nutrition transition.

## Supplementary Information

**Additional file 1.** CONSORT 2010 checklist of information to include when reporting a cluster randomised trial*.

**Additional file 2.** Package insert of multi-vitamin mineral supplement tablets.

## Data Availability

The datasets used and/or analyzed during the current study are not publicly available since the study is still ongoing. Data is available on request from the authors upon completion of the trial.

## Abbreviations

BIA: Bioelectrical impedance analysis
BMI: Body mass index
DBM: Dual burden of malnutrition
FFM: Fat-free mass
FM: Fat mass
HAZ: Height-for-age *z*-score
HV: Height velocity
LMICs: Low- and middle-income countries
NCD: Non-communicable disease
SES: Socio-economic status
TrFFM: Truncal fat free mass
TrFM: Truncal fat mass
WHO: World Health Organization
