# Supplementary material for: Impact of a school-based health intervention program on body composition among South African primary schoolchildren: results from the KaziAfya cluster-randomized controlled trial
Source: BMC Med. 2022 Jan 27;20:27. doi: 10.1186/s12916-021-02223-x (PMC8793158; doi:10.1186/s12916-021-02223-x)
Supplement: Supplementary file 1 — Additional file 1. CONSORT 2010 checklist of information to include when reporting a cluster randomised trial*. [file 12916_2021_2223_MOESM1_ESM.pdf]

CONSORT 2010 checklist of information to include when reporting a cluster randomised trial*

Section/Topic	Item No	Checklist item	Reported on page No
**Title and abstract**			
	1a	Identification as a cluster randomised trial in the title	1
	1b	Structured summary of trial design, methods, results, and conclusions (for specific guidance see CONSORT for abstracts)	2-3
**Introduction**			
Background and objectives	2a	Scientific background and explanation of rationale: **Rationale for using a cluster design**	5-6
	2b	Specific objectives or hypotheses: **Whether objectives pertain to the cluster level the individual participant or both**	5
**Methods**			
Trial design	3a	Description of trial design (such as parallel, factorial) including allocation ratio: **Definitions of cluster and description of how the design features apply to the cluster.**	7-8
	3b	Important changes to methods after trial commencement (such as eligibility criteria), with reasons	NA
Participants	4a	Eligibility criteria for participants **and clusters**	6-7
	4b	Settings and locations where the data were collected	6
Interventions	5	The interventions for each group with sufficient details to allow replication, including how and when they were actually administered: **Whether interventions pertain to the cluster level the individual participant or both**	7-8
Outcomes	6a	Completely defined pre-specified primary and secondary outcome measures, including how and when they were assessed**: Whether outcome measures pertain to the cluster level the individual participant or both**	9-10
	6b	Any changes to trial outcomes after the trial commenced, with reasons	NA
Sample size	7a	How sample size was determined: **Method of calculation, number of clusters and cluster size.**	7
	7b	When applicable, explanation of any interim analyses and stopping guidelines	9
Randomisation:			
Sequence generation	8a	Method used to generate the random allocation sequence	7
	8b	Type of randomisation; details of any restriction (such as blocking and block size): **Detail of stratification if used**	7
Allocation concealment mechanism	9	Mechanism used to implement the random allocation sequence (such as sequentially numbered containers), describing any steps taken to conceal the sequence until interventions were assigned: **Specification that allocation was based on clusters rather than individuals and whether allocation concealment (if any)**	7-8

		**was at the cluster level, the individual participant level, or both**	
Implementation	10a	**Who generated the random allocation sequence, who enrolled participants, and who assigned participants to interventions.**	7-8
	10b	**Mechanisms by which individual participants were included in clusters for the purposes of the trial**	7-8
	10c	**From whom consent was sought (representatives of the cluster, or individual cluster members) and whether consent was sought before or after randomisation.**	6 & 7
Blinding	11a	If done, who was blinded after assignment to interventions (for example, participants, care providers, those assessing outcomes) and how	7-8
	11b	If relevant, description of the similarity of interventions	7
Statistical methods	12a	Statistical methods used to compare groups for primary and secondary outcomes	9-10
	12b	Methods for additional analyses, such as subgroup analyses and adjusted analyses	9-10
**Results**			
Participant flow (a diagram is strongly recommended)	13a	**For each group, the numbers of clusters that were randomly assigned, received intended treatment, and were analysed for the primary outcome**	10-11 Figure 1
	13b	**For each group, losses and exclusions for both clusters and individual members after randomisation, together with reasons**	10-11, Figure 1
Recruitment	14a	Dates defining the periods of recruitment and follow-up	10
	14b	Why the trial ended or was stopped	NA
Baseline data	15	A table showing baseline demographic and clinical characteristics for each group	25
Numbers analysed	16	For each group, number of participants (denominator) included in each analysis and whether the analysis was by original assigned groups	10, Table 1
Outcomes and estimation	17a	For each primary and secondary outcome, results for each group, and the estimated effect size and its precision (such as 95% confidence interval)	11-12, Table 2
	17b	For binary outcomes, presentation of both absolute and relative effect sizes is recommended	NA
Ancillary analyses	18	Results of any other analyses performed, including subgroup analyses and adjusted analyses, distinguishing pre-specified from exploratory	12-13, Tables 3-4
Harms	19	All important harms or unintended effects in each group (for specific guidance see CONSORT for harms)	NA
**Discussion**			
Limitations	20	Trial limitations, addressing sources of potential bias, imprecision, and, if relevant, multiplicity of analyses	16
Generalisability	21	Generalisability (external validity, applicability) of the trial findings	16
Interpretation	22	Interpretation consistent with results, balancing benefits and harms, and considering other relevant evidence	13-15

**Other information**			
Registration	23	Registration number and name of trial registry	6
Protocol	24	Where the full trial protocol can be accessed, if available	NA
Funding	25	Sources of funding and other support (such as supply of drugs), role of funders	1, 7

*We strongly recommend reading this statement in conjunction with the CONSORT 2010 Explanation and Elaboration for important clarifications on all the items. If relevant, we also recommend reading CONSORT extensions for cluster randomised trials, non-inferiority and equivalence trials, non-pharmacological treatments, herbal interventions, and pragmatic trials. Additional extensions are forthcoming: for those and for up to date references relevant to this checklist, see www.consort-statement.org.
