# Supplementary material for: Impact of a school-based health intervention program on body composition among South African primary schoolchildren: results from the KaziAfya cluster-randomized controlled trial
Source: BMC Med. 2022 Jan 27;20:27. doi: 10.1186/s12916-021-02223-x (PMC8793158; doi:10.1186/s12916-021-02223-x)
Supplement: Supplementary file 2 — Additional file 2. Package insert of multi-vitamin mineral supplement tablets. [file 12916_2021_2223_MOESM2_ESM.pdf]

Package Insert

16 Brewery Street
ISANDO, 1600
Tel: +27113986904 Fax: +27113925808
www.dsmnutritionalproducts.com

Study title:
Effects of school based physical activity and multi micronutrient supplementation intervention on growth, health and well-being of schoolchildren in three African countries

Product information – Multi-Vitamin Mineral Supplement tablets for children

Batch number BN: IS2049 / IS2080
Packed: 03/2019 Expiring date: 03/2020

Chewable tablets for oral application.
The multivitamin and mineral tablets are used for clinical trials only.
The multivitamin and mineral tablets are not to be sold or administered to children other than those involved in the trial.
Trial Registration No.: ISRCTN29534081
Principal investigator, Tanzania: Marceline Finda, Ifakara,
Health Institute (IHI), Ifakara, PO Box 53, Tel: +255 232 625164, Fax: +255 232 625312, lfinda@ihi.or.tz

Content of the chewable tablets:

Nutrient	Average per 1 Tablet1
ß-carotene (as BetaTab 20%S)	3.6 mg
Vitamin D	400 IU / 10 mcg
Vitamin E	9 mg TE
Vitamin K	30 mcg
Vitamin C	60 mg
Vitamin B1	1.1 mg
Vitamin B2	1.3 mg
Vitamin B6	0.5 mg
Vitamin B12	1.2 mcg
Folic Acid	200 mcg
Niacinamide	8 mg
Iron (added as Fe-EDTA)	10 mg
Zinc (added as Zinc Oxide)	5 mg
Selenium (added as Sodium Selenite Anhydrous)	20 mcg
Iodine (added as Potassium Iodate)	100 mcg
Orange Flavor	12 mg
Sugar as Sucrose	426.330 mg
Sorbitol	Q.S.

Package Insert

16 Brewery Street
ISANDO, 1600
Tel: +27113986904 Fax: +27113925808
www.dsmnutritionalproducts.com

Placebo:

Specification	Target UoM
Riboflavin Powder as SD	0.20 mg
Cocoa Powder	2.40 mg
Sugar as Sucrose	426.00 mg
Sorbitol	320.00 mg
Citric Acid Anhydrous	40.00 mg
Orange Flavor	10.00 mg

Stability and Storage
In it's original and unopened container, this product may be stored for 12 months from the date of manufacture at a temperature of 25°C (maximum 65% RH). The "Best Before" date is indicated on the label. Keep package tightly closed. Once opened, use the contents quickly.

Safety information
Avoid ingestion and direct contact with eyes by using suitable protective measures. For full safety information and necessary precautions, please refer to the respective Safety Data Sheet (SDS)

Packaging information
Packed and stored in strong containers that are impervious to light and humidity. The containers must be received with an unbroken seal.
